# Defining the value proposition in diagnostic technology: challenges and opportunities for its understanding and development – a review with a multiperspective reflective analysis

**DOI:** 10.3389/fmed.2025.1498618

**Published:** 2025-02-20

**Authors:** Tayana Soukup, Bernarda Zamora-Talaya, Shayan Bahadori, Rosario Luxardo, Patrick Kierkegaard, Omar Butt, Hannah Kettley-Linsell, Katerina-Vanessa Savva, Massimo Micocci, Shanshan Zhou, Simon Newman, Simon Walne, Christopher J. Peters, Adam Gordon, Melody Ni, Peter Buckle, George B. Hanna, NIHR HRC IVD PPIE Team

**Affiliations:** ^1^Department of Surgery and Cancer, Faculty of Medicine, Imperial College London, London, United Kingdom; ^2^Cancer Research UK Convergence Science Centre at The Institute of Cancer Research, London, and Imperial College London, London, United Kingdom; ^3^Royal National Orthopaedic Hospital NHS Trust, London, United Kingdom; ^4^Wolfson Institute of Population Health, Queen Mary University of London, London, United Kingdom; ^5^Academic Centre for Healthy Ageing, Barts Health NHS Trust, London, United Kingdom

**Keywords:** value proposition, diagnostics, digital health, stakeholder perspectives, conceptual framework

## Abstract

**Background:**

The Value Proposition (VP) in diagnostic technology serves as a “positioning statement” outlining the unique benefits, costs, and differentiation an innovation under development offers to healthcare organizations and its ability to effectively deliver these advantages in comparison to current interventions in the market. Despite its significance however, VP lacks a universally accepted definition, which is compounded by the diversity of technologies, their applications, and the varying needs of stakeholders. This paper aims to address this gap by offering a detailed conceptual analysis, revised definition of VP, and actionable recommendations for advancing VP development.

**Methodology:**

We conducted a targeted narrative review, focusing on literature explicitly defining VPs in diagnostic technologies. Using Ovid’s Medline and Embase databases, we identified 19 relevant papers, of which only 5 provided explicit VP definitions. Our analysis incorporated principles of team science, encompassing reflective and thematic analyses of (1) interdisciplinary co-author discussions enabling us to weave together diverse insights into a cohesive exploration of the topic, and (2) MTech’s publicly available set of anonymised responses from NHS Associates, to capture the perspectives of the decision-makers and further enhance depth and breadth of our discourse.

**Results and discussion:**

Our findings highlight the multifaceted nature of VP and its primary hurdles: inadequate identification of unmet needs and insufficient recognition of key stakeholders. We synthesized the evolution of VP definitions and explored the importance of unmet needs in their development, guided by frameworks, such as the Health Technology Navigation Pathway Tool, to ensure VPs meet both the pragmatic and aspirational goals of the healthcare. Thematic insights revealed opportunities for addressing these barriers through implementation science and collaborative strategies. This multi-perspective approach provided a conceptual examination of VP, enabling integration of varied viewpoints and insights.

**Conclusion:**

By employing team science principles and reflective analysis, we introduced a revised definition of VP and a set of actionable recommendations to guide VP development in diagnostics. These findings highlight the importance of addressing stakeholder diversity, unmet needs, and the intricacies of blending interdisciplinary perspectives to advance the field.

## Introduction

A Value Proposition (VP) in healthcare is considered a “positioning statement” that outlines the unique benefits, costs, and differentiation an innovation under development offers (in terms of improved patient-centered care, quality, and effectiveness) to healthcare organizations and its ability to effectively deliver these advantages in comparison to current interventions in the market. As innovations evolve, their corresponding VPs must also adapt to meet the needs of increasingly diverse stakeholders. This adaptability is often a key prerequisite for securing funding, investment, and support for researchers, SMEs, and MedTech developers. Despite its significance however, the concept of VP lacks a universally accepted definition in the context of diagnostic technologies. This ambiguity is further compounded by the diversity of technologies available, their applications, and the varying needs of stakeholders (1, 2).

This manuscript explores the broader category of diagnostic technologies, encompassing both traditional and digitally enabled tools, to address the challenge of defining VPs. The focus on diagnostics reflects their unique role in VP development, particularly their primary role in supporting clinical decision-making and care pathways, with their contributions to health outcomes often being indirect or mediated through subsequent clinical actions. Clarifying these conceptual foundations is essential to establishing a stronger basis for the development of operational models in the future.

While productivity, health outcomes, and care efficiency are critical considerations in applied VP frameworks, this manuscript focuses on conceptual elements foundational to defining VPs, rather than presenting a comprehensive evaluative tool. This focus allows for the identification of how VPs are described and understood across stakeholder groups, serving as a precursor to formalized operational frameworks.

Although this manuscript references UK-based frameworks such as the NHS Health Technology Navigation Pathway (HTNP) and the NHS Accelerated Access Collaborative, these models are provided as illustrative examples due to their structured approaches to VP development. The insights presented aim to be conceptually transferable across multiple healthcare systems where stakeholder engagement and value-based decision-making influence the adoption of diagnostic technologies.

A clearer definition of VPs in diagnostic technologies is expected to provide clarity and a standardized understanding that can streamline innovation development, improve stakeholder alignment, and ensure more effective adoption of diagnostic solutions to meet clinical, operational and economic needs.

Our aim was to offer a comprehensive and reflective analysis of VP development focused exclusively on diagnostics and paying special attention to the diverse challenges and potential opportunities presented from multiple stakeholder viewpoints. Our objectives were to:

1. Examine the evolving definitions of VP in diagnostic technology.
2. Identify and address unmet needs crucial for effective VP understanding and development.
3. Synthesize diverse viewpoints from stakeholders, including NHS clinicians, industry experts, and patient representatives.
4. Explore challenges and opportunities in VP understanding and development for diagnostic technologies.
5. Provide actionable insights and future directions for advancing VP understanding and development, focusing on stakeholder collaboration and adaptability.

## Examining definitions of VP in diagnostics (O1)

We conducted a targeted narrative review to identify papers defining VP in the context of diagnostic technologies. Addressing the lack of a universally accepted definition is critical, as this gap poses challenges for innovators, healthcare providers, and other stakeholders when attempting to advance VP concepts and ensure their practical applicability.

To meet this goal, studies were included if they clearly defined or elaborated on a definition of a VP for diagnostic technologies. Papers that discussed VPs without providing a standalone definition, or those focused solely on broader frameworks rather than explicit definitions, were excluded. This approach ensured that the results offered a robust conceptual foundation for understanding and developing VPs in diagnostics.

Using Ovid’s Medline and Embase databases, we combined the search terms “value propos*” (858 hits) and “diagnos*” (6,340,821 hits) to ensure direct relevance of VPs to diagnostic technologies. This combined search yielded 138 results, subsequently narrowed to 132 English-language publications. After removing duplicates, 129 papers remained, of which five explicitly defined VP (3–7), and two discussed challenges in developing a VP (1, 2). An additional 12 papers elaborated on a VP for a diagnostic technology (8–19). Thus, among these 19 VP-focused papers, only five offered explicit definitions (see Table 1) (3–7). The remaining publications were excluded for not meeting the specified criteria.

**Table 1:** Definitions of value proposition found in literature.

Source	Domain	Definition of value proposition
Lanning and Michaels (1988)	Business	“a clear, simple statement of the benefits, both tangible and intangible, that the company will provide, along with the approximate price it will charge each customer segment for those benefits” [first definition of VP in the literature]
Lehoux et al. (2012)	Medical devices	“…A definition of the value that medical devices bring to health care should acknowledge the complexity and multiplicity of the stakeholders’ perspectives at play and of the dimensions to which they are likely to be responsive”
Price et al. (2014)	Laboratory medicine	“… provides information to enable clinicians to make better decisions about the care of patients.” “… has to recognize (i) the complexity of healthcare, (ii) that benefits accrue elsewhere in the system, reflecting the unmet needs of several different stakeholders, and (iii) the impact of adopting the proposition on each individual stakeholders, and their individual incentive to change.”
Price et al. (2016)	Laboratory medicine	“…the value proposition for laboratory medicine (whether it be overall service or individual test utility) is expressed in terms of contributions to guide decision making in clinical care, the process of the care delivered, and the resource required to deliver that care.” “… is the link between the provider and the needs of the customer. It describes the utility of the product or service in terms of benefit to the customer.”
Graziadio et al. (2020)	Point of care testing	“…We use “value propositions” (plural) with the ordinary language use to mean benefits, whether they are monetary or not.”

We selected Medline and Embase for their strong biomedical and healthcare focus. PubMed’s content largely overlaps with Medline, and Embase complements this coverage by capturing European and industry-related research. Omitting broader interdisciplinary databases like Scopus or Web of Science maintained the diagnostic focus, ensuring a targeted and conceptually pertinent dataset.

Drawing on the selected papers, we identified following key insights. The concept of VP was initially defined by Lanning and Michaels in 1988 (3) and has since been adopted in the commercial sector to “deliver better value.” Table 1 outlines the definitions of VP within the domains of laboratory medicine and medical devices. The authors concurred on the concept’s multifaceted nature, highlighting elements such as clarity, specificity, complexity, and the incorporation of multiple stakeholder perspectives (4–7). They pointed out that at the core of VP should be the benefits—both tangible and intangible—that stem from the adoption of new technology. Furthermore, VP is articulated in ways that support decision-making in clinical care (4, 5), moving beyond merely a product-centric view to highlight the extensive influence of laboratory medicine on the healthcare ecosystem.

Various authors (1, 2), address the complexities involved in crafting a successful VP, despite not defining VP directly. They stress the importance of thoroughly investigating the issues and needs of potential customers and involving a wide array of stakeholders in the process. Furthermore, they assert that identifying unmet needs is crucial for determining the added value brought by innovation in diagnostics (1, 2). Similarly, Rodriguez-Manzano et al. (78) highlight regulatory hurdles and the need to navigate evolving policy landscapes, further illustrating the multifaceted challenges that innovators face in developing effective VPs.

We identified 12 papers (8–19) discussing VPs for a variety of diagnostic technologies. A consensus emerged on the necessity to articulate unmet needs and define clinical, operational, and economic outcomes, as well as highlighting the technology’s more obvious benefits. Three studies (17–19) mention VPs without adhering to a particular framework or covering only selected VP aspects. While these contributions may not encompass all VP development stages, they offer valuable insights and a foundation for further exploration into VPs, as suggested by the authors of these studies (17–19).

While we identified that the primary challenges in developing a successful VP stem from inadequately addressing a clear unmet need and a failure to identify key stakeholders, these barriers can also be viewed as facilitators. In implementation science, it is understood that identifying and understanding barriers to implementation provides an opportunity to develop targeted strategies that can facilitate successful implementation (20–22). One systematic review (20) highlighted the importance of understanding both barriers and facilitators for successful innovation delivery, while another (22) emphasized the importance of context-dependent nature of these factors in the implementation process. By aligning our strategies accordingly, we can convert the challenges into opportunities for VP development, leading to user-centered diagnostics with a higher chance of successful implementation and sustainment.

## Importance of identifying unmet needs in the development of VP (O1, O2)

Some authors (5) emphasized the importance of understanding unmet needs as foundational to defining the VP. Here, we delve deeper into how their identification and understanding are crucial for developing effective VPs that meet both practical and aspirational healthcare goals. Three categories of unmet needs are identified in the literature: clinical, operational, and economic/efficiency (8–19). Recognizing and conceptualizing these needs are essential steps that precede the crafting of the VP and analysis of the care pathway, even when some evidence (e.g., economic and operational unmet needs) is not available during the creation phase (6).

**Figure 1:**
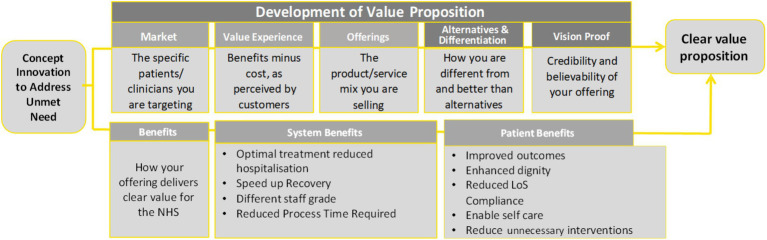
Value proposition analysis based on the Health Technology Navigation Pathway tool, developed by the NHS England Accelerated Access Collaborative (23).

In England, the recently developed Health Technology Navigation Pathway (HTNP) Tool (23), (Figure 1), an initiative of the NHS Accelerated Access Collaborative is designed to guide health technology innovators in integrating new technologies into the NHS framework. As part of the Accelerated Access Collaborative Pathway, it focuses on “affordable products which can dramatically improve efficiency, fill an unmet need or make a step change in patient outcomes” (23). Therefore, the identification of an unmet need is a first step; as well as a recognized key challenge in the formulation of VP (3–5).

Figure 1 illustrates the development of the VP and its elements within the HTNP tool, including system and patient benefits, which are recognized as elements of value in various Value Assessment Frameworks (VAFs).

### Enhancing diagnostic equity: navigating clinical, operational, and economic challenges

The development and integration of health technologies, as exemplified by the HTNP Tool (Figure 1) (23), demonstrates the potential of diagnostics to significantly impact patient care by addressing unmet needs. However, despite widespread recognition of their importance, diagnostics often receive insufficient appreciation in terms of reimbursement and health expenditure. Recent work by Rodriguez-Manzano et al. (78) emphasizes that these systemic challenges are compounded by complex regulatory frameworks, pointing to the critical role of policy alignment and streamlined pathways for the successful integration of diagnostics into healthcare systems. This disparity is a key factor contributing to limited access to diagnostic services (24). The 76th World Health Assembly on strengthening diagnostics capacity (25), identified an urgent need “to consider health technology assessment systems for the systematic evaluation of the effectiveness and cost-effectiveness of diagnostics to support decision-making for the selection of diagnostics for interventions for universal health coverage.” However, the economic assessment of diagnostics within HTA has been less comprehensive compared to therapeutics, particularly in measuring value. As of February 2024, the Cost-Effectiveness Analysis (CEA) Registry (26) recorded 5,573 articles on pharmaceutical interventions but only 877 on diagnostics, highlighting this discrepancy.

A recent systematic literature review summarized evidence on 57 VAFs (27). Notably, the ISPOR Special Task Force (STF) on US Value Assessment Frameworks (28), agreed on elements of value measured in conventional CEA (survival, HRQoL, net costs) and other novel elements of value such as those related to uncertainty, in particular, insurance value, real option value, the value of knowing, and the value of hope (29). These key uncertainty-related elements were included as elements of value of complementary diagnostics (30), and recognized as the first version of the ‘ISPOR value flower’ (31). The review of HTA consideration of elements of value for complementary diagnostics was presented in Zamora et al. (32), including the English Diagnostic Assessment Program (DAP) (33). Zamora et al. (32) highlighted that the NICE DAP acknowledged a broad concept of the novel element “value of knowing,” and how incremental innovation in diagnostics for cancer biomarkers generated “scientific spillovers” as a novel element of value.

The most distinctive novel element of value for diagnostics is the “value of knowing” generated by prognostic and predictive diagnostic information that reduces uncertainty for the patient. The updated NICE manual (34) implicitly recognizes the value of knowing as a clinical outcome, by adopting the DAP broader definition of diagnostic outcomes: “*Relevant outcomes include any health outcomes resulting directly or indirectly from any technologies being evaluated. They may also include informational outcomes of value to the patient for the relief, or imposition, of anxiety or for personal planning”* (34). A review (35) of 53 HTA guidelines across the world reported that value of knowing (reduction of uncertainty) was only included by HTA bodies in 3 countries: Australia, Canada, and Denmark. The recent VAF for *In-Vitro* Diagnostics in Asia Pacific (36), mentions that the Australia’s Medical Services Advisory Committee (MSAC) recommended funding genetic testing for cardiomyopathies considering this novel element of value. In parallel, the London School of Economics collaborative value framework, discussed in Augustovki et al. (76) targets NGS/CGP diagnostics, reinforcing the trend toward comprehensive frameworks that inform VP development by integrating multiple elements of value across diverse healthcare contexts.

Since HTA for diagnostics is mostly limited to measuring short-term health outcomes and costs, innovators may not have incentives to include novel elements of value, such as value of knowing. The relevant health outcomes resulting directly from the innovation are less recognized for diagnostics than for therapies as noted in a recent Augustovski’s et al. systematic review: “*It is difficult in these technologies to translate the evidence into the decision”* (37). Notably, the framework developed by Augustovski et al. (37) emerged from a Latin American context and integrated a multistakeholder deliberative process, emphasizing that defining value for diagnostics may require a different set of metrics than those used for therapeutics. This VAF aligns with other evolving frameworks that collectively push the conceptual boundaries of value assessment for diagnostics beyond traditional cost-effectiveness measures.

The wider value of diagnostic information is initiated in the test and channeled to societal benefit through changes in decision-making for the clinical pathway, with impact on clinicians, carers, and broader society and environment (36, 37, 38). As sustainability and carbon footprint become increasingly important in healthcare decision-making – aligning with Environmental, Societal and Governance (ESG) principles – the potential for diagnostics to contribute to environmental stewardship is gaining attention. Recognizing this dimension expands the assessment of value beyond patient and health system benefits to include the environmental impact of diagnostic development, use and disposal. Recent discussions, as noted by Augustovski et al. (76), emphasize incorporating sustainability metrics into VPs, ensuring that environmental value – such as reduced carbon footprints, resource conservation, and eco-friendly manufacturing processes – is transparently considered.

Also, the value of unmet needs for diagnostics can be considered as included in the novel element of value “equity” Is included as a novel element of value (31), environmental sustainability can also be integrated into VAFs. This ensures that diagnostic innovations are evaluated not only on their clinical, operational, and economic merits but also on their capacity to align with ESG goals, ultimately promoting a more holistic assessment of their value.

### Frameworks for assessing unmet needs

To our knowledge, there is not an established “unmet need framework,” but we use this terminology to refer to broader studies on evidence requirements to support diagnostics development and implementation, even if these studies define elements beyond unmet needs. We have selected and reviewed three “unmet need frameworks” recognized by diagnostic organizations. Two of them include an analysis of interviews with stakeholders across the healthcare ecosystem.

The framework, POCKET (39), points out that: “[…] establishing whether there is a clinical need for a test to be available at the point of care should ideally precede device development to maximize the chances of success.” POCKET presents a checklist that goes beyond unmet needs to all evidence generation needed for implementation, which must be evaluated along the clinical pathway. The second framework from the Working Group of the European Federation of Clinical Chemistry and Laboratory Medicine (EFLM TE-WG) (40, 41) highlights wider aspects of unmet need taking into account the impact on clinical, operational and economic outcomes, similarly to the VP framework for laboratory medicine (5, 15). The third and most recent framework focused on clinical needs due to lack of approved devices or by offering a clinically meaningful advantage over existing approved devices (42). In recognizing that the adoption of innovations relies on the support of all stakeholders, we suggest considering ‘stakeholder buy-in’ as an additional unmet need or outcome in VPs. For instance, equitable healthcare provision and patient access alone do not ensure patient uptake.

### Unmet needs considered relevant by stakeholders

The POCKET checklist (39) has been developed and consulted with four group of stakeholders: clinicians, commissioners and regulators, methodologists and industry. A study on unmet medical device needs for patients with rare diseases (42) surveyed clinicians, mostly physicians and associated FDA advisory committees on rare diseases, separating diagnostics from therapeutic devices. In both, the POCKET and the FDA checklists, the items related to the evidence requirements/unmet needs linked to the clinical pathway overlap. In particular, the definition of evidence requirements to define clinical needs related to the indication, population, and setting, are considered relevant by all stakeholders. POCKET also adds some dimensions related to the clinical pathways that require evidence on consequences - advantages and disadvantages - for the patient and at an institutional or regional level. The consequences for the patient are considered relevant for all stakeholders, but methodologists do not require evidence at institutional or regional levels. The FDA unmet needs study for rare diseases acknowledges barriers in government regulations as a dimension of need, necessary to tackle which is related to the evidence requirement on consequences at institutional and regional levels, as included in the POCKET clinical pathway dimension.

The POCKET (39) and the FDA (42) unmet needs frameworks also consider economic/efficiency needs, although the requirements for a full cost-effectiveness model are only considered necessary for commissioners and regulators in POCKET, whereas all stakeholders consider the information on costs relevant. Profitability for the industry and costs of development are also considered in the FDA unmet needs framework. Of note, all stakeholders consider the required evidence on health outcomes as consequences for the patient. Therefore, some form of health economic evaluation can include costs and health outcomes. For instance, a cost-consequences model can demonstrate that the diagnostic device has the potential to be cost effective even if not aggregating outcomes as quality-adjusted life years (QALYs).

Lastly, the operational unmet needs identified by the laboratory medicine working groups, including those outlined in the most recent toolkit (43), include maintenance, staff training, reassignment costs, system integration, and interoperability. While the FDA does not define such needs, it covers some of their limitations (e.g., turnaround time: “takes too long”) (42).

## Value proposition through the lens of different stakeholder perspectives (O3)

The literature (1, 7), and recent discussions in the field, such as Powell and Hannah (77), emphasize the importance of identifying and understanding of stakeholders’ perspectives as foundational to defining the VP, including, clinical, patient and public, industry and human factors. To begin unpacking these perspectives, we formed an expert group of co-authors who are working in the fields of MedTech/Med devices and come from diverse disciplinary backgrounds, including, clinical, patient and public, industry and human factors. To enhance our interdisciplinary collaboration and dialogue, we embraced the science of team science (SciTS) principles (44, 45), aligning our approach with established big-team science models (46, 47). This was particularly useful in tackling the complex and interdisciplinary nature of VP, enabling us to weave together diverse insights into a cohesive exploration of the topic. To streamline the convergence of our diverse interdisciplinary viewpoints and manage the intricacies involved in blending such varied perspectives (48, 49), we used reflective analysis in, and thematic analysis of our own discussions, geared toward a conceptual examination of VP in diagnostics in terms of what value means and what are the challenges faced.

To enrich the depth and breadth of our discourse, we utilized and thematically summarized publicly available (50) anonymised survey responses from the NHS Associates (e.g., directors, nurses, managers, public health consultants), which explored what value means to NHS decision-makers. These were part of MTech’s (50) engagement strategy aimed at understanding NHS customers’ challenges and decision drivers, crucial for developing effective market access strategies and VPs in the UK healthcare sector.

In what follows, we provide a summary of common concerns and stakeholders’ perspectives, identified through our reflective and thematic exploration, further illustrating how diverse viewpoints shape VP development (77). Their overview is presented in Figure and Table 2, with a description of each perspective provided in Supplementary File 1.

**Table 2:** Common concerns and unique perspectives of stakeholders on value propositions in diagnostic technologies.

Category	Discourse
A. Common concerns	
Adaptability as a strategic imperative	All stakeholders emphasized the importance of diagnostic technologies being adaptable to evolving healthcare landscapes and patient needs, highlighting a dynamic and flexible approach to VP development.
Accessibility	Focusing on the usability aspects of diagnostic technologies, overcoming barriers to use, such as interface complexity and accessibility issues, ensuring that technologies are user-centric and cater to a wide range of abilities.
Clinical effectiveness and economic efficiency	Stakeholders prioritized diagnostics being clinically effective and cost-efficient, advocating for VPs that clearly articulate the health and economic benefits, ensuring technologies provide value for money.
Patient-centeredness	Importance of diagnostics offering comfort, convenience, and addressing psychological impacts, with a focus on patient-centric approaches in VP development.
Integration with existing workflows	The need for diagnostics to seamlessly integrate into existing clinical workflows, enhancing rather than complicating healthcare delivery processes.
Sustainability and environmental impact	Highlighting the sustainability of diagnostic technologies and their environmental impact, reflecting a move toward greener practices in healthcare.
Data security and privacy	Emphasizing the importance of ensuring data security and privacy in the development and implementation of diagnostic technologies, especially in the context of digital health advancements.
Equity and inclusion	The necessity for diagnostic technologies to serve diverse populations adequately, addressing health disparities and promoting equity and inclusion in healthcare outcomes.
B. Unique perspectives (*N* = 39; 5 overlapped)	
NHS Associates (*n* = 17)	System improvements, cost effectiveness, integration within healthcare frameworks, emphasis on evidence-based validation, real-world examples, and equity in healthcare delivery, highlighting the need for diagnostics to demonstrate system-wide benefits.
Clinicians (*n* = 4)	Focus on reliability, speed, accessibility, accuracy, and the need for organizational support to facilitate diagnostics’ integration and operation within healthcare infrastructure, acknowledging diagnostics’ critical role in patient care.
Industry experts (*n* = 3)	Importance of demonstrating economic, operational, and people-focused value, alongside navigating healthcare systems to align product development with unmet needs, addressing the broader context of value in diagnostics.
Patient representatives (*n* = 5)	Personal impact of diagnostics on experiences, emphasizing non-invasive methods, support for autonomy and dignity, and the need for diagnostics to be user-friendly, fast, accessible, and understandable for diverse patient groups.
Human factor specialists (*n* = 5)	The critical role of ergonomic design, ease of use, and user-friendly interfaces in enhancing diagnostic adoption, advocating for a thorough examination of these aspects during the developmental phase to ensure accessibility and simplicity for all users.
Academic researchers (*n* = 10)	Emphasizing the necessity for diagnostics to incorporate evidence-based models, integrating patient data, clinical evidence, and economic analyses for methodological rigor and practical applicability. Highlighting the dynamic nature of value propositions, necessitating iterative development and stakeholder engagement.

NHS Associates comprised following roles, as reported by MTech Team (50) GP Partner, Program Director, Advanced Nurse Practitioner, Program Manager, Clinical Director, PCN Leader, PCN Manager, Trust Finance Lead, Social Care Director, Independent Consultant in Public Health, Commercial Director, Practice Manager, Former Clinical Lead for Medicines Optimization, Head of Integrated Care, Chief Nurse, Head of Medicines Optimization, GP Lead.

**Figure 2:**
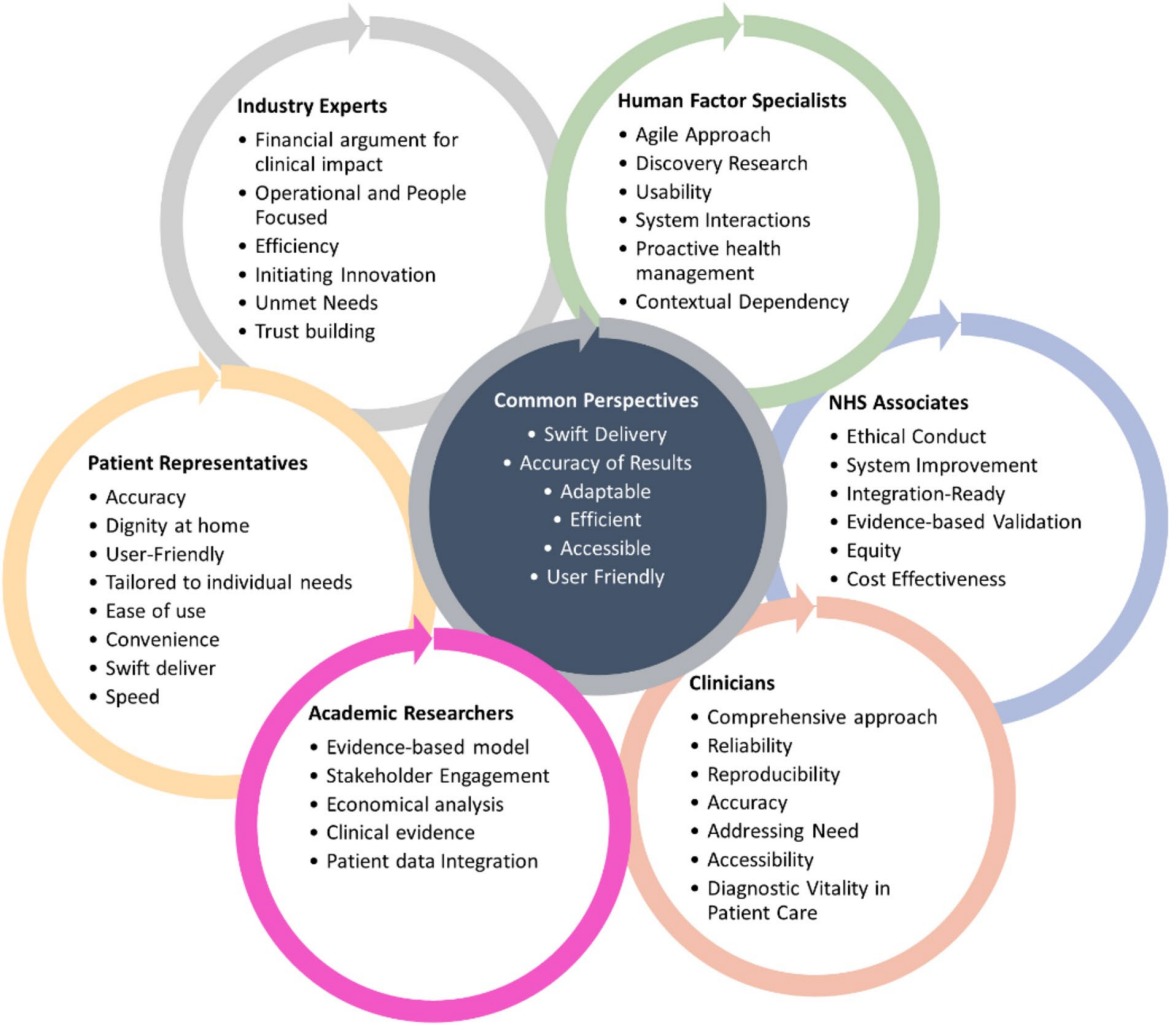
Diagram representing key words and reflections from diverse stakeholders on what value means to them. The themes presented were derived from stakeholder perspectives and do not represent a formalized framework for value proposition development.

Nonetheless, we acknowledge a limitation: while laboratory technicians are important end-users of diagnostic technologies, their perspectives were not captured by either our interdisciplinary co-authors’ reflections or the NHS Associates’ survey data. Consequently, their viewpoint does not appear in Figure 2. Future explorations should explicitly include laboratory technicians’ insights to further enrich the conceptual landscape of VPs in diagnostic technologies.

### Adaptability as a strategic imperative

At the heart of discourse emerged the principle of adaptability. The emergence of diagnostic technologies is a critical factor in advancing healthcare outcomes, necessitating a flexible approach to their integration within the existing healthcare infrastructure (51). However, the rapid pace of change, both in terms of disease profiles and healthcare delivery models, requires diagnostic solutions that are not just reactive but anticipatory. This adaptability is not a technical requirement but a strategic imperative, enabling healthcare systems to remain resilient in the face of emerging challenges and opportunities (52). Hence, the value of diagnostic technologies extends beyond basic functionality, to improving how patients feel about their care, and to enhancing system delivery and sustainability through adaptability. The dynamic nature of diagnostic technologies is a testament to the sector’s commitment to evolving alongside the healthcare needs of the population, ensuring that advancements in diagnostics are both reflective of and responsive to the shifting landscape of healthcare demands.

### Accessibility

In addressing the critical aspect of usability in diagnostic technology development, a nuanced understanding of specific challenges such as interface complexity and accessibility issues emerged as essential. Methods such as participatory design, where end-users are involved in the development process, and agile development frameworks that allow for rapid iteration based on user feedback, have proven effective in overcoming these hurdles. Such strategies ensure that technologies are not only technically proficient but also user-centric, catering to a wide range of abilities and reducing barriers to effective use (19, 39).

### Balancing clinical effectiveness with economic efficiency

The dialogue around clinical effectiveness and economic efficiency encapsulated a dual focus that is critical in the context of constrained healthcare budgets and the imperative for high-quality care. The nuanced balance between cost and benefit, articulated through frameworks such as those employed by NICE (34), highlights a sophisticated understanding of value that extends beyond monetary metrics to encompass health outcomes and quality of life (37). This perspective represents a maturing healthcare ecosystem that seeks to optimize resource allocation, ensuring that the adoption of diagnostic technologies is both a clinically and economically judicious decision.

### Patient-centeredness

In the development and implementation of diagnostic technologies, patient-centeredness was fundamental, representing a shift toward more holistic and empathetic healthcare delivery (53). The emphasis on patient experience—comfort, convenience, and psychological impact—signals a move away from a one-size-fits-all approach to diagnostics, toward solutions that are tailored to the diverse needs and preferences of patients. This shift not only enhances the diagnostic technologies but also reinforces the centrality of the patient in healthcare decision-making processes.

### Integration with existing workflows

It was clear that the importance of diagnostics seamlessly integrating into existing clinical workflows cannot be overstated, because it is crucial for enhancing rather than complicating healthcare delivery processes (54). Successful adoption hinges not only on clinical efficacy and cost-effectiveness (24) but also on the technology’s compatibility with healthcare professionals’ established routines (36). Minimizing disruption, maximizing user acceptance, and fully realizing the benefits of these technologies in practice are essential (52). Effective strategies for achieving this integration encompass the development of intuitive interfaces (29), comprehensive training for healthcare staff (43), and ensuring new technologies align with existing protocols and systems (55). By focusing on the seamless integration of diagnostic technologies into clinical workflows, improvements can be achieved in operational efficiency, patient outcomes, and creating a more resilient system (53).

### Sustainability and data security

Emerging themes such as the sustainability of diagnostic technologies and the imperative for data security reflect an expanding understanding of value that incorporates environmental awareness and the ethical use of patient data (11, 43). These considerations, alongside the foundational principles of equity and inclusion, highlight the multifaceted nature of value in diagnostics, and an interplay between technological advancement and societal good.

### Equity and inclusion

Accessibility emerged as a critical axis around which the discourse on diagnostic technologies orbits. Specifically, the equitable distribution of healthcare resources, including diagnostics, is a barometer for the inclusivity of healthcare systems (24). In bridging the gap between advanced healthcare settings and remote or underserved areas, diagnostic technologies act as a lever for equity, ensuring that every individual, irrespective of geography or socioeconomic status, has access to the benefits of medical advancements. This focus on accessibility not only addresses the practical challenges of healthcare delivery but also has a broader commitment to universal health coverage, a cornerstone of global health initiatives (36).

### Navigating unique stakeholder perspectives for shared value

The dialogue around VP development in diagnostics was further enriched by the unique perspectives of various stakeholders. From NHS associates’ (50) focus on system-wide benefits and integrated care to clinicians’ emphasis on reliability and accuracy, each perspective contributed to a composite view of value that is as diverse as the ecosystem it seeks to serve (18, 56). Industry experts navigate the complexities of market access and product development, while patient representatives and human factors specialists advocate for diagnostics that are not only effective but also humane, accessible and inclusive (1, 8, 39).

Exploring the intersections and potential conflicts between stakeholder perspectives reveals a complex mix of challenges in aligning around a shared definition of value. For instance, the industry’s drive for innovation and market penetration can sometimes clash with healthcare providers’ focus on cost-effectiveness and clinical utility. Similarly, the push for state-of-the-art diagnostic solutions may inadvertently sideline user-friendly design principles, highlighting the tension between technological advancement and accessibility. Navigating these complexities requires a concerted effort to foster dialogue and compromise, ensuring that the development and implementation of diagnostic technologies truly reflect the collective interests and values of all stakeholders (43, 56).

In synthesizing these diverse perspectives, it becomes clear that the future of diagnostics lies not just in their technical capabilities but in their integration within a broader healthcare, societal, and ethical framework. As we look toward the future, the challenge and opportunity for stakeholders across the healthcare spectrum is to collaboratively navigate this complex landscape, highlighting the power of diagnostic technologies to not only advance healthcare outcomes but also to embody the values of adaptability, accessibility, efficacy, inclusivity, security and sustainability.

## Discussion

### Navigating complexities and enhancing measurements (O4)

Advancing the VP in diagnostics presents exciting opportunities for innovation. For example, precision and personalized medicine, supported by genomics and advanced technologies, tailors diagnostics to individual needs (57). Digital health platform integration enhances monitoring and remote access (58), while AI/ML contribute toward improved diagnostic accuracy and offer predictive analytics for proactive healthcare (59). Point-of-care testing delivers crucial rapid results in remote or underserved areas (60), and multi-modal diagnostics combine imaging, lab tests, and clinical data for comprehensive health assessments (61).

However, VPs, being dynamic and context-dependent, require an inclusive, collaborative development approach that aligns with existing frameworks and involves extensive stakeholder engagement, beyond the immediate team (62, 63). A patient-centered diagnostic approach, in particular, can broaden the focus to include satisfaction, usability, access, and its role in the care continuum, treating healthcare value as a complex construct (64). An effective VP harmonizes diverse, sometimes conflicting, stakeholder priorities, crafting diagnostics that are clinically and economically sound, patient-focused, ethical, and well-integrated into the healthcare system (65).

Recognizing diagnostics’ multidimensional nature—clinical, economic, technological, and ethical—further highlights the need to address stakeholder values comprehensively including those of patients, providers, insurers, and technology manufacturers (55, 64, 65). Evidence-based models that incorporate patient data, clinical evidence, and economic analyses are essential in ensuring methodological rigor and applicability, supported by empirical research and stakeholder feedback for continuous VP refinement (31). VP needs to evolve with changing stakeholder needs and technological advancements, maintaining their relevance and effectiveness, with clear articulation of the proposition providing balance between value demonstration and proprietary information (54).

Core to this strategy are psychometrically robust measurement systems, providing comprehensive value assessments with both quantitative and qualitative insights into stakeholder perspectives (66), embracing SciTS principles for enhanced interdisciplinary collaboration (44, 45). Applying implementation science methods further enhances VP development and refinement by identifying adoption barriers and ensuring diagnostics’ sustainability and adaptability meet evolving healthcare needs (67). Such psychometrically sound system is also essential for accurately assessing VP success, while addressing the broad needs and expectations to enable more precise diagnostic innovation evaluation (66, 68, 69). It is important to adapt across diseases and innovations to provide a consistent value evaluation method in varied contexts, aiding decision-making and resource allocation by highlighting VP similarities and differences across diseases and innovations (70). This structured system should also prioritize patient-centered outcomes (e.g., quality of life and accessibility), reflecting the broad spectrum of stakeholder priorities and enhancing the framework’s overall impact (71).

### Future needs in the field and recommendations (O5)

The diagnostic landscape within the UK’s NHS has undergone significant transformations, shaped by a complex interplay of fiscal constraints, resource allocation, and shifting healthcare priorities (51). These transformations have variably affected different medical conditions, emphasizing the inherent complexities and disparities within diagnostics (56). The integration of novel technologies and evolving policy frameworks has further complicated this landscape, leading to an uneven distribution of diagnostic resources that often disproportionately benefit certain conditions, such as cancer, compared to others, such as mental health (72).

For example, the UK’s Innovative Devices Access Pathway (IDAP) pilot phase aims to support innovative technologies and solutions to address unmet clinical needs (73). It narrows indications and target population to potentially life-threatening or seriously debilitating conditions, but the criteria require evidence on system wide benefit, including for adoption and sustainability and cost-effectiveness. Therefore, any medical device applying to enter this pilot phase must formulate a VP to cover an unmet clinical need.

Furthermore, it is arguable that stakeholders buy-in need should be considered as a relevant dimension not recognized before, as preceding VP. In the unmet needs frameworks (39–42), patients were not among the stakeholders consulted, and only the POCKET framework (39) included needs that are key to achieve uptake of diagnostics: acceptability as “their attitudes to test (including how this was determined).” The FDA framework (42) included a limitation of a diagnostics test as “invasive, cumbersome, painful and/or inconvenient.” Only the POCKET (39) included all stakeholders’ views as an evidence requirement in the form of stakeholder analysis (identification of individuals/groups likely to be affected by test adoption, the impact of adoption and their attitudes). This evidence requirement or unmet need by an existing diagnostic must be also demonstrated by any innovator to fulfill the IDAP criterion, i.e., “The product will be widely adopted and is sustainable” (73).

Central to the effectiveness of a VP in diagnostics is its dual capacity to address current unmet needs while also anticipating future healthcare demands. This deep understanding is vital for aligning VPs with both the practical realities of healthcare delivery and aspirational goals of enhanced patient care and diagnostic innovation (73). In developing these propositions, ensuring their validity and reliability to reflect the diverse values and emerging trends across stakeholder groups is crucial. Such a robust VP not only meets technical and psychometric standards but also embodies empathy and foresight, effectively bridging the gap between today’s service provisions and tomorrow’s healthcare aspirations (53).

Addressing the present gaps in diagnostic VPs necessitates a focused trajectory in future research. There is an urgent need for greater inclusivity in stakeholder representation and for VPs to adapt to the dynamic healthcare landscape. Future research should focus on creating dynamic, yet psychometrically robust, VPs that can accommodate shifts in healthcare priorities and technological advancements, utilizing implementation science. Enhancing stakeholder engagement methods (e.g., with SciTS principles) and integrating advanced data analytics and AI into the VP development process are critical areas warranting attention (52). This advancement will enable the development of effective VPs in diagnostics, meeting the healthcare system’s diverse needs (74, 75).

Building upon this article’s insights, we propose eight actionable recommendations for developing comprehensive and strategic VPs for diagnostic technologies, aligned with NICE’s aims and approach (34), addressing both the immediate and long-term needs of the healthcare system (Table 3). These recommendations span from identifying unmet needs to ensuring sustainability and impact, culminating in our refined VP definition:

**Table 3:** Actionable recommendations for value proposition development in healthcare diagnostics aligned with aims and approach of NICE HT evaluation.

Recommendation	Action	Alignment with Aim and Approach of NICE HT Evaluation
1. Identifying and addressing unmet needs	Focus on identifying and addressing unmet needs in healthcare diagnostics to ensure that the VP comprehensively responds to current and anticipated challenges.	Directly supports A1 by fostering the rapid and consistent adoption of innovative diagnostics, addressing gaps that hinder such adoption. It also aligns with A2 and A3 by targeting improvements in treatment decisions and resource utilization.
2. Multidisciplinary stakeholder engagement	Enhance the involvement of a wide range of stakeholders, incl. Patients, healthcare providers, and policymakers, to ensure a holistically informed VP development.	Enriches A1 through A3 by ensuring that a wide range of perspectives are considered, which is vital for the adoption and practical application of diagnostic technologies.
3. Economic and ethical analysis	Expand the economic evaluations and ethical analyses to include broader societal impacts, ensuring the VP balances cost-effectiveness with wider moral, societal and ethical considerations.	Aligns with the NICE HT approach by ensuring that cost considerations are balanced with ethical imperatives, contributing to the comprehensive cost-minimization strategy.
4. Evidence-based integration with implementation science	Strengthen the use of evidence-based models integrating clinical, economic, and patient data, while applying implementation science methods to identify barriers and design tailored implementation strategies.	Supports A2 and A3 by applying evidence-based approaches to improve clinical decision-making and NHS resource efficiency through the implementation of scientifically validated diagnostic technologies.
5. Dynamic adaptability and implementation science	Emphasize the continuous refinement of the VP based on new research and multidimensional feedback, using implementation science frameworks to proactively adapt to evolving healthcare needs and technological advancements.	Enhances the NICE HT evaluation process by ensuring that VPs remain relevant over time, meeting A1’s goal of consistent adoption while adapting to emerging challenges.
6. Effective communication strategy	Refine and diversify communication strategies to articulate the VP’s multifaceted value and relevance to all stakeholders, ensuring enhanced comprehension, engagement, and collaboration.	By communicating the value effectively, this recommendation aims to improve the adoption rates (A1), informed treatment choices (A2), and resource utilization (A3).
7. Building a knowledge base through measurement and psychometrics	Further develop the VP’s accuracy and reliability by focusing on advanced measurement and psychometric methods, thereby creating a more robust and responsive knowledge base.	Complements the NICE HT cost-minimization approach by enhancing the reliability of VPs, ensuring that cost analyses are underpinned by accurate and validated measures.
8. Prioritizing sustainability and long-term impact with implementation science	Focus on sustainability and long-term impact in VP development, employing implementation science to strategically navigate and adapt to the evolving nature of healthcare systems and technologies.	All 3 NICE HT aims by ensuring that VPs not only address immediate needs but are also sustainable and consider long-term impacts on the health system.

NICE HT evaluation aims: A1. Promote the rapid and consistent adoption of innovative clinically and cost-effective diagnostic technologies in the NHS. A2. Improve treatment choice or length and quality of life by evaluating diagnostic technologies that have the potential to improve key clinical decisions. A3. Improve the efficient use of NHS resources by evaluating diagnostic technologies that have the potential to improve systems and processes for the delivery of health and social care. NICE HT Evaluation Approach: App1. Cost-minimisation approach to assess products costs and resource consequences resulting from, or associated with, the technology under evaluation and comparator technologies. App2. It considers clinical benefits (for example, effectiveness outcomes) alongside the cost analysis.

*‘A VP in diagnostics is a dynamic, evidence-based statement that articulates the specific and measurable benefits a diagnostic technology offers all healthcare stakeholders. It reflects a comprehensive understanding of unmet needs, aligning with both operational efficiencies and economic value. It adapts to healthcare advancements and market demands, addressing the requirements of varied healthcare systems while responding to multifaceted challenges. It highlights the innovation’s value in improving clinical outcomes, system efficiency, and stakeholder benefits in a complex ecosystem.*’

This definition encapsulates the essence of the article’s insights and recommendations, serving as a guiding roadmap for advancing VPs in diagnostics. However, it is pertinent to acknowledge that while this article explores various perspectives, the exploration’s breadth and depth have limitations. The breadth of exploration could benefit from the inclusion of more professional groups (e.g., commissioners) across the national spectrum to capture a wider array of experiences and viewpoints. Similarly, by engaging with a larger number of stakeholders, a deeper, more nuanced understanding of the ecosystem could be achieved. Recognizing the potential to expand our insights through broader and deeper engagement highlights the foundational nature of our current work, helping unveil layers of insight that might otherwise remain obscured. Such endeavors not only enrich the discourse on VPs but also pave the path for future directions, enhancing the scope and effectiveness of diagnostic technologies in the healthcare ecosystem.

## Limitations

This review employed a targeted narrative approach, focusing on literature that explicitly defined VPs in diagnostic technologies. While this provided conceptual clarity, it narrowed the scope of our findings. By prioritizing explicit definitions over broader thematic inquiry, we may have excluded potentially informative perspectives or indirectly relevant frameworks that could offer additional context. Future research could adopt a more expansive approach, incorporating a wider range of methodologies and literature sources.

Furthermore, the stakeholder perspectives captured in our thematic and reflective analyses were drawn from our interdisciplinary team of co-authors and publicly available NHS Associates’ survey responses. While this approach aligned with the principles of team science and facilitated the integration of multiple viewpoints, it inadvertently omitted certain key stakeholders, notably laboratory technicians. Their exclusion limits insight into operational and technical considerations vital to VP implementation. Future efforts should incorporate laboratory technicians to further enrich the conceptual landscape of VPs in diagnostics.

The NHS frameworks cited in this manuscript, including the HTNP and Accelerated Access Collaborative, serve primarily as illustrative models rather than prescriptive frameworks. While these tools are referenced to demonstrate structured Value Assessment Frameworks approaches, with the VP at the creation phase, the insights presented aim to be conceptually transferable across multiple healthcare systems where stakeholder engagement and value-based decision-making influence the adoption of diagnostic technologies.

Additionally, this manuscript does not include real-world examples of diagnostics with established VPs. This decision was intentional, as the primary focus is on advancing conceptual clarity around the foundational elements of VPs rather than assessing applied models. While constructs such as productivity, health outcomes, and care efficiency are critical components in practical VP models, many cannot be included in the VP creation phase until more evidence is generated. The primary aim was to clarify definitional elements rather than prescribe an exhaustive set of criteria for VP assessment. Future work could extend this conceptual foundation by exploring how clearly defined VPs influence the adoption and sustainability of diagnostic technologies in applied contexts, where metrics such as productivity, health outcomes, and care efficiency could be more systematically incorporated and evaluated along evidence generation.

Despite these limitations, our application of team science principles demonstrates a replicable and adaptable methodology that can be extended to larger, more diverse stakeholder cohorts. By doing so, future investigations can achieve a more nuanced, equitable, and comprehensive understanding of the multifaceted elements shaping VPs in diagnostics.

## Conclusion

We embarked on an analytical journey to unravel the complex dynamics of VP development in diagnostics, shedding light on the nuanced challenges and opportunities that define this process. Drawing on a literature search, while utilizing the principles of team science, we highlighted the importance of meeting unmet needs and fostering engagement across varied stakeholders. The focus on diagnostic technologies in this manuscript was intentional, providing foundational clarity on how VPs are described, which may inform broader operational models for both diagnostic and other health technologies in future work. Our findings advocate for a flexible and refined approach to VP formulation, one that is in harmony with the fast pace of innovation and transformation in the healthcare and technological sectors. Through this lens, our paper serves as a cornerstone for future endeavors aimed at optimizing the integration and impact of diagnostic innovations, ultimately contributing to the advancement of patient care and system efficiency.
