# Supplementary material for: Defining the value proposition in diagnostic technology: challenges and opportunities for its understanding and development – a review with a multiperspective reflective analysis
Source: Front Med (Lausanne). 2025 Feb 20;12:1498618. doi: 10.3389/fmed.2025.1498618 (PMC11884263; doi:10.3389/fmed.2025.1498618)
Supplement: Supplementary file 1 [file Data_Sheet_1.docx]

# SUPPLEMENTARY FILE 1: THEMATIC ANALYSIS OF INDIVIDUAL STAKEHOLDER GROUPS

## 4.1 Reflections of NHS associates on what value means and what are the challenges faced

[The MTech team](https://mtechaccess.co.uk/what-does-value-mean-to-nhs-decision-makers/) (2022) gathered insights from NHS Associates through surveys and discussions at the NHS Engagement Symposium in 2022. The aim was to understand the priorities in the NHS regarding treatments and technologies, focusing on the challenges and decision-making drivers that are essential for crafting successful UK market access strategies and VPs. They highlighted the importance for industry stakeholders to grasp the NHS's definition of 'value' and to communicate effectively with NHS decision-makers. Understanding this definition of 'value' becomes crucial when considering the methodologies employed by organizations such as NICE in their health technology evaluations. The NICE HT Evaluation approaches the assessment of costs and benefits of products, proposing different methods of health economic evaluation, mainly cost-effectiveness to measure cost per quality-adjusted life years (QALYs) which can be added by a new technology over the standard of care. The evaluation process aims to ensure that the technologies recommended for adoption offer value for money by balancing the broad benefits and costs of the provision of health services or social care. Other criteria are also considered by NICE, including equity. In what follows, we present the key themes from the MTech’s (2002) interviews with NHS Associates, offering an in-depth look at the insights and implications drawn.

On the question of ‘*What Value Means to You?*’, NHS Associates (n=17) reported it as multifaceted, encompassing ethical conduct, cost-effectiveness, patient experience, system improvements, teamwork, leadership, and clinical outcomes, all within the context of constrained resources. Specifically, then perceived value as being treated with empathy, kindness, and adherence to the seven Nolan principles of managerial conduct. These aspects are seen as markers of quality in healthcare, rather than fitting into a traditional market model. They also perceived value in terms of ensuring money is spent well, focusing on the patient rather than specific organizations or clinicians. It is not about providing care for less than a fixed sum but about maximizing the effectiveness of expenditures with the patient as the focus. They value an improved patient experience and making a positive difference to patients. This includes tackling health inequalities and involving patients in their care, which aligns with the concept of value as a unit of community. Value is also reported to be found in improved systems and processes that enhance the capacity and capabilities of services without adding workload or detrimentally impacting patient health or experience. This improvement is seen as freeing up time for teams, especially given workforce challenges, and supporting an integrated approach across systems. It is seen as associated with improved workforce experience, inclusivity, respect, and the impact of one's role within the team or workplace. Kindness, leadership, culture, and language were reported as core to the stakeholders' collective needs, emphasizing the non-commercial nature of clinical care. Lastly, they defined value in terms of improved quality of care and outcomes. The NHS is expected to place value on the care it offers, which cannot be limitless and needs to be clearly defined and communicated. Hence delivering the best possible care within constrained resources is as such seen as a critical aspect of value (MTech, 2022).

In the response to the question on ‘*What Would You Need to Hear From Industry to Make You Sit Up, Listen and Take Action?*', the NHS Associates reported seeking proof that healthcare products and innovations are clinically effective, enhancing systems and processes, and offering good value for money. They are interested in real-world examples demonstrating the positive outcomes of these innovations, including their impact on patient care and the potential for cost savings, while ensuring that any new implementations do not adversely affect the healthcare workforce. The stakeholders were particularly keen on solutions that can deliver financial savings, increase efficiency, and provide significant benefits to patients, all while being financially sustainable for the NHS (MTech, 2022).

## 4.2 Clinicians reflections on what value means and what are the challenges faced

The development of a VP for diagnostic tests in healthcare necessitates a comprehensive approach that considers a spectrum of factors, including reliability, reproducibility, accuracy, cost-effectiveness, and the broader impact on the healthcare system (Bobelyn et al., 2023).

From a clinical perspective (n=4), prioritizing factors such as test speed, accuracy, and accessibility is paramount. Speed is critical for timely decision-making in urgent conditions, while accuracy is vital in high-stakes health situations (Fritz et al., 2022). The ease of interpreting test results also plays an essential role, guiding clear clinical decisions. Moreover, the accessibility of tests, particularly in urgent scenarios, coupled with cost-effectiveness, are pivotal, often serving as major barriers to adoption. This is exemplified in the field of point-of-care solutions, where the immediacy of access and economic viability are crucial (Bobelyn et al., 2023; Fuller et al., 2021). Cost considerations can limit access to affordable testing solutions, highlighting the need for budget-conscious healthcare approaches. In this context, the integration of diagnostics, especially for critical health concerns such as early cancer detection, becomes a prime example of the need to balance these factors effectively (Froelich et al., 2022; Graziadio et al., 2020). Additionally, the importance of these barriers is further emphasized in microbiology, where point-of-care testing is pivotal for patient outcomes (Hansen, 2020).

In the UK's socialized healthcare setting, cost-effectiveness is a crucial determinant for successful implementation, balancing the test's cost against its benefits to the NHS (Oellerich et al., 2020). This emphasis differs from private healthcare settings, where direct patient value might be more prioritized (Garrison Jr. & Austin, 2007; Towse & Garrison, 2017). Strategies such as retesting or using the test as a trial tool can mitigate accuracy issues, aligning with resource constraints (Hansen, 2020; Price et al., 2016).

The role of organizational support in the provision of effective diagnostic tools is critical. It ensures the seamless integration of these tests into various medical contexts, addressing potential systemic bottlenecks and facilitating improved patient management. This organizational aspect is essential in ensuring the tests' successful adoption and operation within the healthcare infrastructure (Froelich et al., 2022).

Moreover, the VP of a diagnostic test extends to its impact on patient management. Its role in early diagnosis, such as in cancer detection, can lead to better outcomes and more efficient healthcare delivery. However, this necessitates a responsive healthcare system capable of handling the outcomes of the diagnostic test to avoid overloading services, inefficiencies, and ensure effective patient care (Augustovski et al., 2021).

The value of a diagnostic test is also vital in its early development process. Concerns arise when academics focus on tests based on convenience or available historical data, often overlooking the practical applicability in intended clinical settings such as GP surgeries. This approach contrasts with the priorities of startup investors, who tend to focus more on the potential impact and value of the test over intricate scientific and practical details (e.g., Vellido, 2020).

Stakeholder belief, including that of patients, GPs, and hospital doctors, is crucial for the successful adoption of innovations including diagnostic tests (e.g., Boon et al., 2023). Overcoming barriers to acceptance, such as scepticism about new technologies or concerns about cost and accessibility, is essential. These factors are instrumental in ensuring that diagnostic tests are not only developed but are also effectively integrated into clinical practice.

The intricacies of multi-test diagnostics, encompassing health economics (cost-benefit analyses, risks of false positives/negatives), and broader system-level impacts, are significant in diagnostic decision-making. Challenges in multi-disease diagnostics include complexities in decision-making and potential organizational strain. Effective communication and interpretation of test results are essential for shared decision-making among healthcare professionals, patients, families, and multidisciplinary teams. To maximize perceived value, developing an individualized VP for tests, tailored to both patient and healthcare professional needs, is critical.

## 4.3 Human factors reflections on what value means and what are the challenges faced

From the human factor’s perspective (n=5), the contextual dependency of a diagnostic test's value is a fundamental point in developing a VP. This context includes existing clinical pathways and the diverse requirements of different user groups, including the necessity of considering a multitude of factors and perspectives when evaluating the value of diagnostic tests in the healthcare context (di Ruffano et al., 2012).

The significance of an agile approach in the development of diagnostic tests needs to be stressed (di Ruffano et al., 2012). This approach involves quick discovery research to swiftly identify and adapt to changes in the perceived value of the test. Moreover, the healthcare providers have the pivotal role in shaping the perceived value of a diagnostic test (Froelich et al., 2022). Moreover, healthcare providers have the pivotal role in shaping the perceived value of a diagnostic test (Hansen, 2020). Our experience shows that the approval and active use of these tests by healthcare providers can substantially influence their perception within the healthcare system.

The subjectivity of value emphasizes that the perception of a diagnostic test's value can vary significantly among different user groups, age demographics, and individual experiences. From our perspective, human factors considerations, such as usability and system interactions, are vital in influencing the perceived value of diagnostic tests. We advocate for a thorough examination of these aspects during the developmental phase (e.g., Huddy et al., 2019).

Additionally, we observe the evolving role of patients who are increasingly proactive about their health, which may significantly influence their perception of the value of diagnostic tests. We acknowledge that the heavy workloads faced by clinicians, particularly due to administrative tasks, can be barriers to the adoption of new diagnostic tests (Greaves et al., 2023). Simplifying their daily activities may be crucial in gaining their acceptance. Finally, the pressure and resource constraints commonly experienced in healthcare settings can significantly shape how healthcare professionals perceive the value of new diagnostic tests (di Ruffano et al., 2012).

## 4.4 Patient and public reflections on what value means and what are the challenges faced?

From the patient and public perspective (n=5), **swift delivery and accuracy** of results are crucial elements in the VP of diagnostics. Extended waiting periods for results, potentially stretching to weeks, not only heighten patient stress but also delay essential treatments (Graziadio et al., 2020). Some individuals may accept a slight degree of inaccuracy (e.g. 5-10%) for the ease and comfort of home testing. This indicates that accuracy, while vital, isn't the only factor in determining the value of these tools. Comfort, convenience, and the ability to maintain dignity at home are also significant in assessing their overall worth (Fuller et al., 2021). Speed is essential, as delays can cause undue anxiety and impede care, making the ability to quickly provide results a highly valued feature (Hansen, 2020). Similarly, when evaluating new diagnostic systems or devices, a balanced consideration of accuracy, speed, and ease of use becomes imperative, each contributing equally to their efficacy and acceptance in the healthcare landscape.

The **cost and accessibility** of diagnostic tools, especially for home use, are crucial factors in their overall effectiveness and appeal. The convenience of using diagnostic tools at GP surgeries, as opposed to enduring long waits at hospitals, adds significant value to patient care. Affordable and reliable home diagnostic tools offer patients, particularly those who are vulnerable or elderly, the comfort of privacy and the option of self-medication, away from hospital settings (Lam, 2020). While affordability is essential, higher costs can be justified for tests designed for specific patient groups, such as those with cultural, religious, disability, or neurodiverse considerations, or for point-of-care tests used at home (Price et al., 2016). Conversely, extremely low costs may raise concerns about the quality and reliability of the test.

The ease of use of the diagnostic tool for everyone (people with mobility issues or disability) and the comprehensibility of the results are also important. Results should be presented in a way that is easy for patients and their loved ones to understand, rather than in a purely clinical sense (Oellerich et al., 2019). For example, simply providing a temperature reading doesn't mean much unless it is put into context. It would be more helpful if the results indicated whether the reading was significantly different from the norm. There is also value in designing diagnostic tools in a way that does not require a tutorial or a lot of explanation. A tool that is simple to use and provides easy-to-understand results would have a higher value (O’Kane et al., 2020).

The ability to easily interpret the results of a diagnostic tool, in a way that can lead to easy interpretation of healthcare needs is important in determining VP. Even for those who are tech-savvy, a tool that is simple to use and does not require a tutorial or extensive explanation is preferred. The ability to interpret results without needing to connect to an application or other device is also valuable (Huddy et al., 2019).

It is important to consider individual needs and preferences. Not everyone will be comfortable or able to use certain technologies, and these individual differences need to be taken into account. For example, some may prefer a non-invasive test due to for example the discomfort and inconvenience often associated with invasive tests (Wrede et al., 2022).

In prioritizing the development and deployment of technology, trust and accessibility are paramount. It is essential that these innovations are not only within easy reach of those who need them but also consistently reliable, delivering accurate results. In the current climate of escalating living costs in the UK, the affordability of these technologies cannot be overlooked. While trust and accessibility take the forefront, other elements such as speed, accuracy, user-friendliness, result interpretation, and tailoring to individual preferences and needs also play crucial roles. These factors, however, may shift in importance depending on specific circumstances and personal perspectives (Fleming et al., 2021; WHO, 2023).

## 4.5 Industry reflections on what value means and what are the challenges faced?

From the industry perspective (n=3), language and knowledge of the health system are often the precursors for the challenge of understanding and providing impactful VPs. Typically, we observe the following when engaging with them and supporting their understanding. Value in the commercial sense is linked to profit or return on investment; therefore, when asked by a health system to demonstrate value most commercial and industrial ventures will default to a financial argument to support their clinical impact. This perspective aligns with the understanding that a business is fundamentally a value delivery system (Lannings & Michaels, 1988; Porter, 2010). Arguably, this is the typical extent to which industry communicates value, a single clinical and often system-wide, not budget-specific financial proposition.

To tackle this, health systems have developed peripheral institutes to support industry innovators, such as the Academic Health Science Networks (AHSNs) in England who develop programmes and process to upskill and inform (Bobelyn et al., 2023). However, despite the development of these offers, there is confusion within these peripheral institutes who all have a different definition of value and a different process for innovators to uncover or develop their VP (Froelich et al., 2022). At the AHSN, the process challenges innovators to view VPs from multiple perspectives but does not validate if the VPs designed will resonate with the target audience. This leads to a core problem with VPs from an industry perspective, which is access to the health system to uncover the stakeholders impacted by a technology and therefore who they need to create VP’s for, and what they need to communicate. Even when industry manages to access a healthcare provider site, it can take months, if not years, to understand the clinical pathway and the staff that facilitate it (Grasiadio et al., 2020).

This is added to further by the need for the innovator to meet and build trust with those individuals in the hope that they will share information, to support alignment of the feature or benefit of an innovation to that individual’s needed value. Variance in healthcare provision and siloed working practices mean that industry must go through this process with every potential customer. This is costly and results in smaller organisations failing to scale and ultimately ceasing to exist (Fuller et al., 2021).

On a separate note, we also observe the following: Industry often fails to understand the link between VP and evidence. They are typically aware that evidence is used for regulatory approval or clinical validation but are unaware of the role agnostic 3^rd^ party validation has on their VPs in de-risking the health system decision to procure (Fritz et al., 2022). The HCPs are wary of “sales” pitches and would rather rely on impact validation from a reputable organisation. They have become wary because of industry creating misleading VPs based on a shallow understanding of how the system works. For example, industry often claims to help the HCPs save money by reducing the number of hours a member of staff will work on a problem. We know that those hours are not real savings as the staff are a fixed cost (Hansen, 2020). We see this type of statement regularly with industry unable to bridge this gap in their understanding with the current provision of resources.

We also regularly observe that industry is unaware of the areas in which VPs need to be created beyond clinical and financial. Operational and people-focused VPs are not common practice, even from large well-established organisations such as the ‘big pharma’ (Lam, 2020). We also see that import and export of innovation faces similar issues and compound confusion of industry over VPs and how best to design, validate and support with evidence (Oellerich et al., 2019; Oellerich et al., 2020).

Finally, we note that Industry lacks knowledge of the HCPs language and will communicate VPs using industry terms or misunderstand system need as a result. For example, if an HCP asks for cash release to be a value of an innovation, few from an industry background will see that this is in their terms the need to demonstrate a return on investment (Price & St John, 2014, 2019).
